# Acupuncture stimulation improves scopolamine-induced cognitive impairment via activation of cholinergic system and regulation of BDNF and CREB expressions in rats

**DOI:** 10.1186/1472-6882-14-338

**Published:** 2014-09-17

**Authors:** Bombi Lee, Bongjun Sur, Jaegul Shim, Dae-Hyun Hahm, Hyejung Lee

**Affiliations:** Acupuncture and Meridian Science Research Center, College of Korean Medicine, Kyung Hee University, 26 Kyungheedae-ro, Dongdaemun-gu, Seoul, 130-701 Republic of Korea; BK21 PLUS Korean Medicine Science Center, College of Korean Medicine, Kyung Hee University, Seoul, 130-701 Korea

**Keywords:** Scopolamine, Memory, Cholinergic neurons, Brain-derived neurotrophic factor, cAMP-response element-binding protein

## Abstract

**Background:**

Acupuncture is an alternative therapy that is widely used to treat various neurodegenerative diseases and effectively improve cognitive and memory impairment. The aim of this study was to examine whether acupuncture stimulation at the Baihui (GV20) acupoint improves memory defects caused by scopolamine (SCO) administration in rats. We also investigated the effects of acupuncture stimulation at GV20 on the cholinergic system as well as the expression of brain-derived neurotrophic factor (BDNF) and cAMP-response element-binding protein (CREB) in the hippocampus.

**Methods:**

SCO (2 mg/kg, i.p.) was administered to male rats once daily for 14 days. Acupuncture stimulation at GV20 was performed for 5 min before SCO injection. After inducing cognitive impairment via SCO administration, we conducted a passive avoidance test (PAT) and the Morris water maze (MWM) test to assess behavior.

**Results:**

Acupuncture stimulation at GV20 improved memory impairment as measured by the PAT and reduced the escape latency for finding the platform in the MWM test. Acupuncture stimulation at GV20 significantly alleviated memory-associated decreases in the levels of choline acetyltransferase (ChAT), BDNF and CREB proteins in the hippocampus. Additionally, acupuncture stimulation at GV20 significantly restored the expression of choline transporter 1 (CHT1), vesicular acetylcholine transporter (VAChT), BDNF and CREB mRNA in the hippocampus. These results demonstrate that acupuncture stimulation at GV20 exerts significant neuroprotective effects against SCO-induced neuronal impairment and memory dysfunction in rats.

**Conclusions:**

These findings suggest that acupuncture stimulation at GV20 might be useful in various neurodegenerative diseases to improve cognitive functioning via stimulating cholinergic enzyme activities and regulating BDNF and CREB expression in the brain.

## Background

Alzheimer’s disease (AD) is a progressive neurodegenerative disorder of the brain that is characterized by deterioration of memory and cognitive function due to cholinergic nervous system dysfunction [1]. Decreased cholinergic function in the brain, as primarily observed in patients with AD, can result in a decline in memory and cognitive function [2]. Accordingly, various cholinergic drugs have been approved to treat or alleviate AD, and they exert their therapeutic effects by counteracting acetylcholine (ACh) deficits and consequently enhancing ACh levels in the brain [3]. In fact, the most common therapy for AD is administration of acetylcholinesterase (AChE) inhibitors, such as donepezil, galantamine, and rivastigmine, which temporarily increase the availability of ACh at cholinergic synapses [4]. Nevertheless, new drugs used to treat AD patients are limited due to their short half-lives and excessive side effects caused by peripheral cholinergic system activation and hepatotoxicity, the most frequent and critical side-effect of these drugs [5]. Thus, an alternative treatment modality for AD patients is required.

Scopolamine (SCO) is a tropane alkaloid drug that exhibits competitive antagonism at muscarinic acetylcholine receptors (mAChRs) by interfering with cholinergic transmission in the central nervous system CNS; [6]. Therefore, SCO administration to animals is used as an experimental model of the cognitive deterioration and memory disturbances in AD; this animal model is frequently used to screen for drugs that have potential therapeutic value in AD-type dementia patients [7–9]. SCO administration not only induces dysregulation of the cholinergic neuronal pathway and memory circuits in the CNS but also reduces the expression of brain-derived neurotrophic factor (BDNF) and cAMP-response element-binding protein (CREB) in the brain; thus, BDNF and CREB may act a novel therapeutics to treat hippocampal dysfunction and memory deficits [10, 11]. BDNF is believed to be responsible for synaptic plasticity and memory performance, particularly in the water maze test, and is coupled to CREB activation [12, 13]. Several studies demonstrated that hippocampal BDNF and CREB are important in long-term memory formation [14], and play important roles in pathological conditions and neurodegenerative diseases such as AD [15, 16].

In East Asian nations, acupuncture is used widely to treat many neurodegenerative disorders, including AD, Parkinson’s disease (PD) and dementia [17]. Its therapeutic effects and mechanism of action have been investigated in both clinical and animal studies. This alternative therapy is known to modulate biochemical balance in the CNS and to maintain homeostasis [18]. Specifically, Baihui (GV20) is one of the most important acupoints targeted to alleviate neurodegenerative disorders and cognitive impairment in acupuncture treatment [19]. Several studies showed that acupuncture stimulation at GV20 reduces cerebral infarct and increases dopamine levels in the brain tissue of ischemia-reperfusion injured rats [20], and reduces the amount of apoptotic neurons in the hippocampal CA1 area of rats with vascular dementia [19]. Although a brief report of the anti-dementia or anti-ischemic activity of GV20 acupuncture stimulation has been published, whether GV20 acupuncture stimulation therapeutic efficacy in alleviating spatial cognitive function following repeated SCO-induced neuronal impairment is due to cholinergic system regulation or BDNF and CREB expression remains unknown [21]. Indeed, one study failed to demonstrate a therapeutic effect of acupuncture on AD [22]. Such major differences could be attributable to differences in acupuncture stimulation parameters, as selection of appropriate stimulation parameters is a crucial factor in the efficacy of acupuncture [23]. Therefore, it is appropriate to investigate changes in spatial cognitive function and neuronal biomarkers in SCO-treated cognitive impairment to better understand the therapeutic effects and mechanisms of action of acupuncture stimulation at GV20.

The aim of the present study was to evaluate the ability of acupuncture stimulation at GV20 to improve learning and memory in rats exposed to repeated SCO-induced memory deficits as measured by their performance in the passive avoidance test (PAT) and the Morris water maze (MWM) test. We also investigated how these activities were related to the cholinergic system and the expression of CREB and BDNF in the CNS, and whether acupuncture stimulation exerted anti-AD activity in this model to elucidate neural mechanisms underlying the memory-enhancing effect of acupuncture stimulation.

## Methods

### Animals

Adult male Sprague–Dawley (SD) rats weighing 220–240 g were obtained from Samtako Animal Co. (Seoul, Korea). The rats were housed in a limited-access rodent facility with up to five rats per polycarbonate cage. The room controls were set to maintain the temperature at 22 ± 2°C and the relative humidity at 55 ± 15%. Cages were lit by artificial light for 12 h each day. Sterilized drinking water and standard chow diet were supplied ad libitum to each cage during the experiments. The animal experiments were conducted in accordance with the National Institutes of Health *Guide for the Care and Use of Laboratory Animals* (NIH Publications No. 80–23), revised in 1996, and were approved by the Kyung Hee University Institutional Animal Care and Use Committee. All animal experiments began at least 7 days after the animals arrived.

### Experimental groups

**Figure 1:**
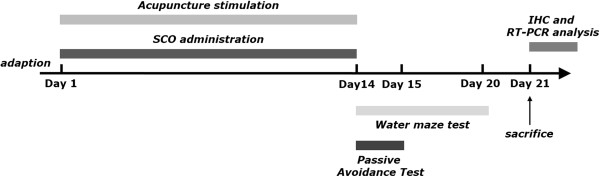
**Experimental schedules for scopolamine (SCO) administration to induce spatial memory impairments in rats.** The experiment was designed to explore the efficacy of acupuncture therapy in healing chronic SCO-induced spatial memory impairment in an animal model using behavioral and neurobiological methodologies. All rats excluding the SAL group received SCO injection. Two weeks after SCO injection, the rats were subjected to the Morris water maze (MWM) and the passive avoidance test (PAT). After behavioral testing, rats were sacrificed and brain tissues was collected immediately. SCO: scopolamine; IHC: Immunohistochemistry.

In order to develop learning and memory deficits in the brain, the rats were intraperitoneally injected with 2 mg/kg SCO, dissolved in physiological saline, once a day for 14 days. Normal animals received saline instead of SCO as a vehicle control. Different rats in an experimental group were subjected to either behavioral testing or immunohistochemistry. The rats were randomly divided into five groups of six or seven individuals as follows: normal group (SAL group, n = 7), the SCO-induced and saline-treated group (SCO group as a control, n = 7), the SCO-induced and Baihui (GV20) acupoint-stimulated group (SCO + GV20 group, n = 7), the SCO-induced and Yangji (TE4) acupoint-stimulated group (SCO + TH4 group, n = 6), and SCO-induced and non-acupoint (on the tail)-stimulated group (SCO + TA group, n = 6) every daily for 5 min before the SCO injection. Scopolamine hydrobromide were purchased from Sigma-Aldrich Chemical Co. (St Louis, MO, USA). The experimental schedule of drug administration, acupuncture stimulation and behavioral tests is shown in Figure 1.

### Acupuncture stimulation

Acupuncture stimulation was bilaterally performed every day for 5 min before the SCO injection during the SCO-injection period. The acupuncture stimulation was performed as previously described [24]. The GV20 acupoint, meeting point on the Governing vessel with the six yang channels, is anatomically located on the midsagittal line, at the intersection of a line connecting the right and left ear apices [25]. The acupoint on tail and the Yangji acupoint were selected as a non-acupoint and a comparison acupoint, respectively. As a comparable control acupoint, we also performed the stimulation to another acupoint, the TE4 acupoint, on hand Shaoyang triple energizer meridian, is located at the midpoint of the dorsal crease of the wrist, in the depression on the ulnar side of the tendon of the extensor muscle of the finger [26, 27]. In addition, the non-acupoint needling was performed at one-fifth point of tail length from the proximal end of the tail to avoid the tail acupoints located at proximal or distal region of the tail. Sterilized disposable stainless steel acupuncture needles (0.30 × 25 mm, Suzhou Kangnian Medical Devices Co., Ltd., Shzhou, China) were inserted perpendicularly as deep as 2–3 mm at GV20 or TE4 acupoint. The depth of needle insertion at each acupoint was arbitrarily determined based on several previously studies [28] and in the animal acupuncture atlas [21]. During the acupuncture procedure, rats were gently handled entirely to minimize stress in the rats. We did not carry out sham acupuncture for control. The SAL and SCO groups were handled for 5 min to a calming effect, instead of acupuncture stimulation, similar to what we described previously [24].

### Passive avoidance test

The test was basically performed according to the step-through method. The Gemini Avoidance System (SD Instruments., San Diego, CA, USA) was used for this experiment. Basically, the step-through passive avoidance apparatus (PAA) consists of a tilting floor acrylic box divided into two-compartments, a lightened compartment connected to a darkened compartments by an automatic guillotine door and a control unit generating an electric shock (Behbood Pardaz Co., Ghaem, Iran). The electric shock can be delivered to the grid floor, made of stainless steel rods (3 mm diameter) spaced 1 cm apart, in both compartments. First, the rats were given trials to acquisition test in the apparatus. In the training session, a rat was placed in a lightened compartment of the PAA facing away from the entrance to the dark compartment, and then the guillotine door was opened. Because of intrinsic preference to the dark environment, the rat immediately entered the dark compartment and the door was closed. During acquisition test, the latency time before entry into the dark compartment was recorded for each rat. After 30 min, the rats were placed in the lightened compartment once again. After entering the dark compartment, the guillotine door was closed and subsequently a mild electrical shock (0.5 mA) was applied for 3 s. The retention test was started 24 h after the acquisition trial for training. The rat was again placed in the lightened compartment and the guillotine door was opened. In the retention test, the rat was placed in the PAA as previously described and the time required for the rat to enter the dark compartment was measured for a maximum period of 3 min in the same method with the acquisition test. The rat did not enter the dark compartment within this period received a latency time of 180 s.

### Morris water maze test

The MWM test was performed in a small circular pool (2.0 m in diameter and 0.35 m deep) made of polypropylene and internally painted white. The pool was half-filled with water to a depth of 30 cm. The water in the pool was made opaque by adding 1 kg skim milk powder and continuously maintained at 22 ± 2°C. The pool was divided into four quadrants of equal area. During the MWM test, an escape platform (15 cm in diameter) was located in one of four sections of the pool, hidden 1.5 cm below the water surface and approximately 50 cm from the sidewalls. Several visual cues were placed around the pool in plain sight of the animals. A digital camera was mounted to the ceiling straight above the center of the pool and was connected to a computerized recording system equipped with a tracking program (S-MART; PanLab Co., Barcelona, Spain), which permitted on- and off-line automated tracking of the paths taken by the rats. The MWM test was initiated on the 14^th^ day after the SCO administration commenced. The animals received three trials per day. The rats were trained to find the hidden platform, which remained in a fixed location throughout the test. The trials lasted for a maximum of 180 s, and the escape latency was expressed by the swimming time to find the submerged platform in the pool. The animals were tested with three trials per day for 5 days, and they received a 60-s probe trial on the sixth day. Finding the platform was defined as staying on it for at least 4 s before the acquisition time of 180 s ended. When the rat failed to find the platform in the limited time in first trial of hidden platform test, the rats should be placed on the platform for 20 s and assigned a latency of 180 s. Between one trail and the next, the water in the pool was stirred to remove olfactory traces of previous swim patterns. The entire schedule proceeded for 6 days and each animal had three trials for training per day with a 40–50 min inter-trial interval. For the probe trial, a rat was placed in the quadrant located diagonally from the target quadrant and allowed to swim to the quadrant from which the escape platform had been removed for a maximum of 60 s. The probe trial was expressed by the ratio of the time spent (or the distance traveled) in searching for the platform in the target quadrant to the total duration spent swimming in the pool.

### Open field test

Prior to water maze testing, the rats were individually housed in a rectangular container that was made of dark polyethylene (60 × 60 × 30 cm) to provide the best contrast to the white rats in a dimly lit room equipped with a video camera above the center of the room, and their locomotor activities (animal’s movements) were then measured. The locomotor activity indicated by the speed and distance of movements was monitored by a computerized video-tracking system using the S-MART program (PanLab Co., Barcelona, Spain). After 5 min of adaptation, the distance they traveled in the container was recorded for another 5 min. The locomotor activity was measured in centimeters. The floor surface of each chamber was thoroughly cleaned with 70% ethanol between tests.

### Immunohistochemistry

For immunohistochemical studies, three rats in each groups were deeply anesthetized with sodium pentobarbital (80 mg/kg, by intraperitoneal injection) and perfused through the ascending aorta with normal saline (0.9%), followed by 300 ml (per rat) of 4% paraformaldehyde in 0.1 M phosphate-buffered saline (PBS). The brains were removed in a randomized order, post-fixed over-night, and cryoprotected with 20% sucrose in 0.1 M PBS at 4°C. Coronal sections 30 μm thick were serially cut through the hippocampus using a cryostat (Leica CM1850; Leica Microsystems Ltd., Nussloch, Germany). The sections were obtained according to the rat atlas of Paxinos and Watson [29]. The primary antibodies against the following specific antigen were used: choline acetyltransferase (ChAT; sheep polyclonal ChAT, 1:2,000 dilution, Cambridge Research Biochemicals Co., Bellingham, UK), acetylcholinesterase (AchE; goat polyclonal AchE, 1:2,000 dilution, Santa Cruz Biotechnology Inc., CA, USA), BDNF (rabbit polyclonal BDNF, 1:200 dilution, Cell signaling., Bostron, MA, USA) and CREB (rabbit polyclonal CREB, 1:250 dilution, Cell signaling., Bostron, MA, USA). Briefly, the sections were incubated with primary antiserum in PBST (PBS plus 0.3% Triton X-100) for 72 h at 4°C. The sections were incubated for 120 min at room temperature with secondary antibody. The secondary antibodies were obtained from Vector Laboratories Co. (Burlingame, CA, USA) and diluted 1:200 in PBST containing 2% normal serum. To visualize immunoreactivity, the sections were incubated for 90 min in avidin-biotin-peroxidase complex (ABC) reagent (Vectastain Elite ABC kit; Vector Labs. Co.), and incubated in a solution containing 3,3’-diaminobenzidine (DAB; Sigma) and 0.01% H_2_O_2_ for 1 min. Finally, the tissues were washed in PBS, followed by a brief rinse in distilled water, and mounted individually onto slides. Images were captured using the AxioVision 3.0 imaging system (Carl Zeiss, Inc., Oberkochen, Germany) and processed using Adobe Photoshop (Adobe Systems, Inc., San Jose, CA, USA). The sections were viewed at 400× magnification, and the numbers of ChAT-, AchE-, BDNF-, and CREB-labeled cells was quantified in the CA1 of hippocampus. The immunopositive cells were counted in at least three different hippocampal sections per rat brain according to the stereotactic rat brain atlas of Paxinos and Watson [29]. The counted sections were randomly chosen from equal levels of serial sections along the rostral-caudal axis. For measuring the number of ChAT, AchE, BDNF and CREB-positive labeled cells were counted only if it reached a defined darkness above background [6]. Lightly immunolabeled cells were not counted. Distinct brown spots indicating ChAT-, AchE-, BDNF- and CREB-immunopositive cells were observed in the cytoplasms and in the membranes of cone-shaped cells in the CA1 of hippocampus. The differences in brightness and contrast among raw images were not adjusted, in order to exclude any possibility of subjective selection of the immuoreactive cells.

### Total RNA preparation and RT-PCR analysis

The expression levels of choline transporter 1 (CHT1), vesicular acetylcholine transporter (VAChT), muscarinic acetylcholine receptor type 1 (mAChR-M1), BDNF and CREB mRNAs were determined by reverse transcription**-**polymerase chain reaction (RT**-**PCR). The brain hippocampus was isolated from three or four rats per each group. The total RNA was prepared from the brain tissues using a TRIzol® reagent (Invitrogen Co., Carlsbad, CA, USA) according to the supplier’s instruction. Complementary DNA was first synthesized from total RNA using a reverse transcriptase (Takara Co., Shiga, Japan). PCR was performed using a PTC**-**100 programmable thermal controller (MJ Research, Inc., Watertown, MA, USA). All primers were designed using published mRNA sequences of those cytokines and a primer designing software, Primer 3, offered by the Whitehead Institute for Biomedical Research (Cambridge, MA, USA; http://www.genome.wi.mit.edu) on the website. The PCR products were separated on 1.2% agarose gels and stained with ethidium bromide. The density of each band was quantified using an image-analyzing system (i**-**Max^TM^, CoreBio System Co., Seoul, Korea). The expression levels were compared each other by calculating the relative density of target band, such as CHT1, VAChT, mAChR-M1, BDNF and CREB to that of glyceraldehyde 3-phosphate dehydrogenase (GAPDH).

### Statistical analysis

All measurements were performed by an independent investigator blinded to the experimental conditions. The results in the figures are expressed as the mean ± standard error of the means (SE). Differences within or between normally distributed data were analyzed by analysis of variance (ANOVA) using SPSS (Version 13.0; SPSS, Inc., Chicago, IL, USA), followed by Tukey’s *post hoc* test. Statistical significance was set at *p* < 0.05.

## Results

### Effect of acupuncture stimulation of GV20 on SCO-induced step-through latency deficit in the passive avoidance test

**Figure 2:**
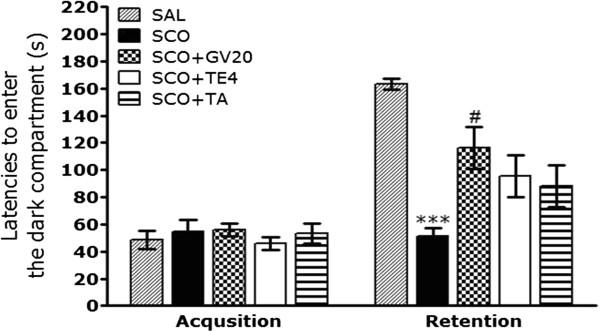
**Effect of GV20 acupuncture stimulation on latency to enter the dark compartment in the acquisition trial and the retention test in the passive avoidance test (PAT).** The rats were randomly divided into five groups of six or seven individuals as follows: normal group (SAL group, n = 7), the SCO-induced and saline-treated group (SCO group as a control, n = 7), the SCO-induced and Baihui (GV20) acupoint-stimulated group (SCO + GV20 group, n = 7), the SCO-induced and Yangji (TE4) acupoint-stimulated group (SCO + TH4 group, n = 6), and SCO-induced and non-acupoint (on the tail)-stimulated group (SCO + TA group, n = 6) every day for 5 min before SCO injection. ^***^ *p* < 0.001 *vs*. the SAL group; ^#^ *p* < 0.05 *vs*. the SCO group.

To determine whether acupuncture stimulation at the GV20 promotes the recovery of memory dysfunction, acupuncture was performed to the rats with SCO-induced impairment of memory, and their memory and cognitive functions were examined by the PAT (Figure 2). It was verified that the rats in all groups had no physiological defect (i.e., motor function defect) or intrinsic cognitive impairment through acquisition trials without electric challenge. During the time for acquisition trials, indicated by the latencies for entering the dark compartment, there were no significant differences among all groups. After acquisition trials, the effect of acupuncture stimulation at the GV20 on the retention latency was examined 24 h after applying electric shock in the dark box in the PAT. In the retention trials, it was shown that in the rats in the SCO + GV20 group had significantly increased latencies to enter the dark compartment for retention as compared to those in the SCO group (*p* < 0.05). This suggests that acupuncture stimulation of GV20, but not of TE4 or the tail, restored memory impairment-related behavior.

### Effect of acupuncture stimulation of GV20 on SCO-induced spatial memory impairment in the water maze test

**Figure 3:**
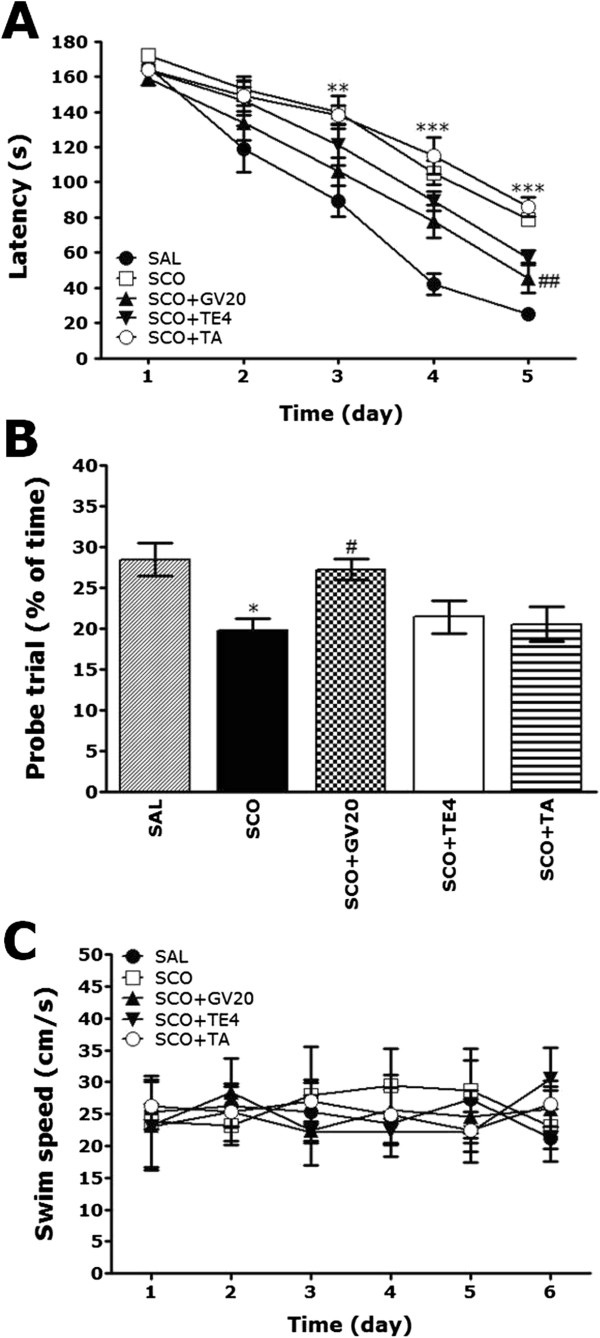
**Effects of GV20 acupuncture stimulation on time to escape (latency) from water during acquisition trials using a submerged platform (A), on the percentages of time in a probe trial without a platform (B), and swim speed (C) in the Morris water maze (MWM) task (n = 6-7/group).** ^*^ *p* < 0.05, ^**^ *p* < 0.01, ^***^ *p* < 0.001 *vs*. the SAL group; ^#^ *p* < 0.05, ^##^ *p* < 0.01 *vs*. the SCO group.

The effect of acupuncture stimulation at the GV20 on swimming to reach the submerged platform in the MWM test is elucidated in Figure 3. Rats in the SAL group rapidly learned the location of the submerged hidden platform and reached it within 20 sec on day 5 of the trials. The SCO group showed marked retardation in escape latency, probably due to memory deficits resulting from SCO-induced impairment of learning and memory. The analysis of escape latency revealed that rats in the SCO + GV20 group had significantly reduced swimming latency as compared with that in the SCO group (*p* < 0.01 on days 5; Figure 3A). To investigate the effect on spatial memory, the performance in the probe trial on day 6 was examined by analyzing the percentages of time spent swimming to the expected position of the platform (Figure 3B). The swimming times were reduced in the rats that swam directly and without confusion to the target area where the platform had been located. The rats with SCO injection showed severe impairment of spatial performance in the MWM test (*p* < 0.05). The rats in the acupuncture stimulation of GV20-treated group spent more time around the platform area than those in the SCO group (*p* < 0.05). The SCO group was not significantly different from the other groups in terms of the mean swimming speed, as calculated by dividing the total swim distance by latency (*p* > 0.05; Figure 3C). Based on these results, rats treated with acupuncture stimulation at the GV20 were suggested to show greater improvement in acquisition during the hidden platform trial and, accordingly, reached the platform quicker than the SCO-treated rats. The results also indicated that the swimming latency of the SCO-induced rats treating acupuncture stimulation of GV20, but not of TE4 or the tail, restored memory impairment-related behavior.

### Effect of acupuncture stimulation of GV20 on SCO-induced motor functions in the open field test

**Figure 4:**
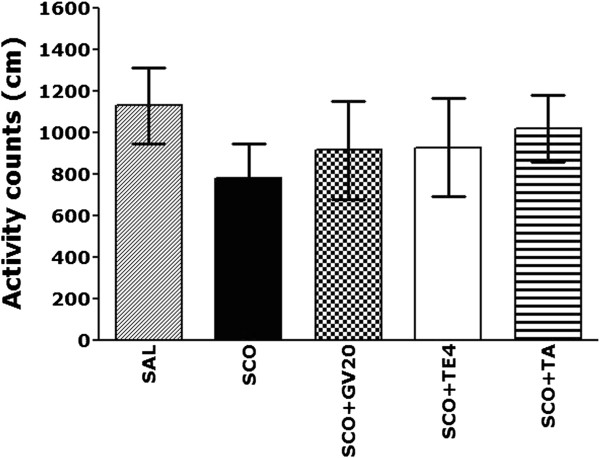
**Locomotor activity counts in the open-field test (n = 6-7/group).**

Open field activity was used to evaluate locomotor activity in the rats that received repeated SCO injection for 14 days (Figure 4). These results indicated that the rats in all groups had no effects on their locomotor activities (motor function) in the open-field test. Because no significant differences in locomotor activities was observed between all groups in the open-field test, it could be suggested that the observed impairment of memory of the rats with repeated SCO injection was not attributed to the differences in their locomotion activities. This may reflect an active response and water avoidance stress when the animal is confronted with a MWM test. However, our results suggest that the rats in all groups displayed no anxiolytic-like behavior in the open-field test after a pretest stress exposure in the MWM test. This indicates that acupuncture stimulation at the GV20 did not affect the active responses or psychomotor function as measured by the rats’ performance in the MWM test.

### Effect of acupuncture stimulation of GV20 on SCO-induced immunohistochemical changes of ChAT, AchE, BDNF and CREB expression in the hippocampus

**Figure 5:**
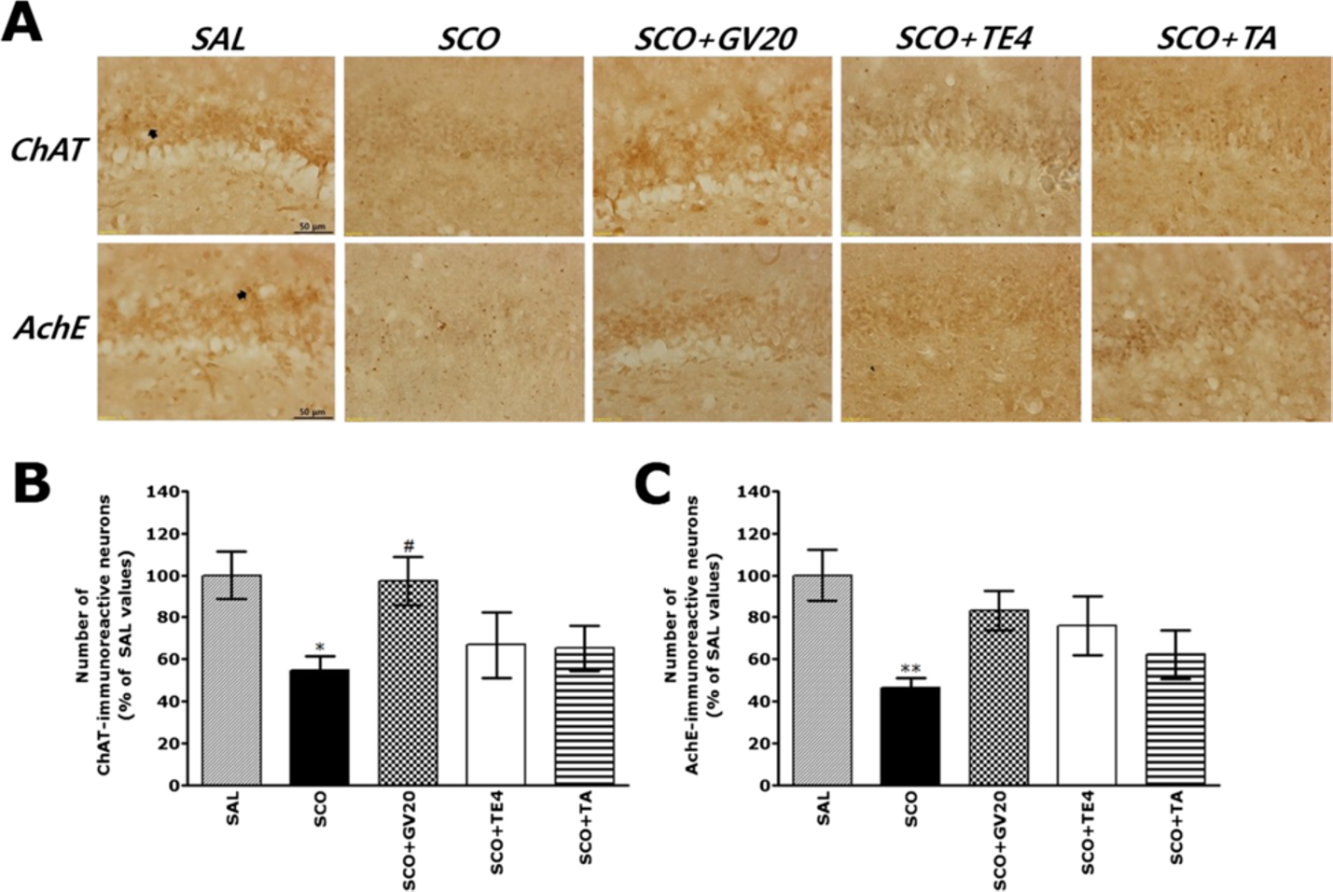
**Effect of GV20 acupuncture stimulation on the percentage values of the mean (±standard errer, SE) number of choline acetyltransferase (ChAT)-stained neurons and acetylcholinesterase (AchE)-stained neurons in various hippocampal areas after the Morris water maze (MWM) task (n = 3-4/group). Representative photographs and the relative percentage values are shown in (A) and (B and C), respectively.** (a): ChAT expression in the SAL group, (b): ChAT expression in the SCO group, (c): ChAT expression in the SCO + GV20 group, (d): AchE expression in the SAL group, (e): AchE expression in the SCO group, (f): AchE expression in the SCO + GV20 group. Sections were cut coronally at 30 μm. Scale bar represents 50 μm. ^*^ *p* < 0.05, ^**^ *p* < 0.01 *vs.* the SAL group; ^#^ *p* < 0.05 *vs.* the SCO group.

Following the behavioral tasks, brain tissue samples from the subjects were analyzed using immunohistochemistry to investigate the effect of acupuncture stimulation at the GV20 treatment on neuronal loss associated with SOC-induced memory impairment. The results of the ChAT and AchE immunoreactivity analysis in the hippocampal cholinergic neuron areas are shown in Figure 5A. The number of ChAT-immunoreactive neuronal cells in the rat hippocampus in the SCO group was significantly decreased compared with that in the SAL group (*p* < 0.05; Figure 5B). The ChAT-reactive neuronal activity in the hippocampus was significantly increased in the hippocampal region in the SCO + GV20 group compared with that in the SCO group (*p* < 0.05). The results also indicated that the SCO-induced rats treating acupuncture stimulation of GV20 was significantly increased ChAT expression in the hippocampal region compared with those in the SCO + TE4 group and the SCO + TA group. Similarly, the number of AchE-immunopositive neuronal cells in the rat hippocampus in the SCO group was significantly decreased compared with that in the SAL group (*p* < 0.01; Figure 5C). The AchE-reactive neuronal activity in the hippocampus associated with SCO-induced memory impairment was not significantly restored in the SCO + GV20 group compared with that in the SCO group (*p* > 0.05). However, rats receiving acupuncture at the acupoint GV20 showed no difference in AchE expression in the hippocampus as compared with those in the controls (SCO group) or rats receiving acupuncture at the nonspecific acupoint tail or TE4 point.

**Figure 6:**
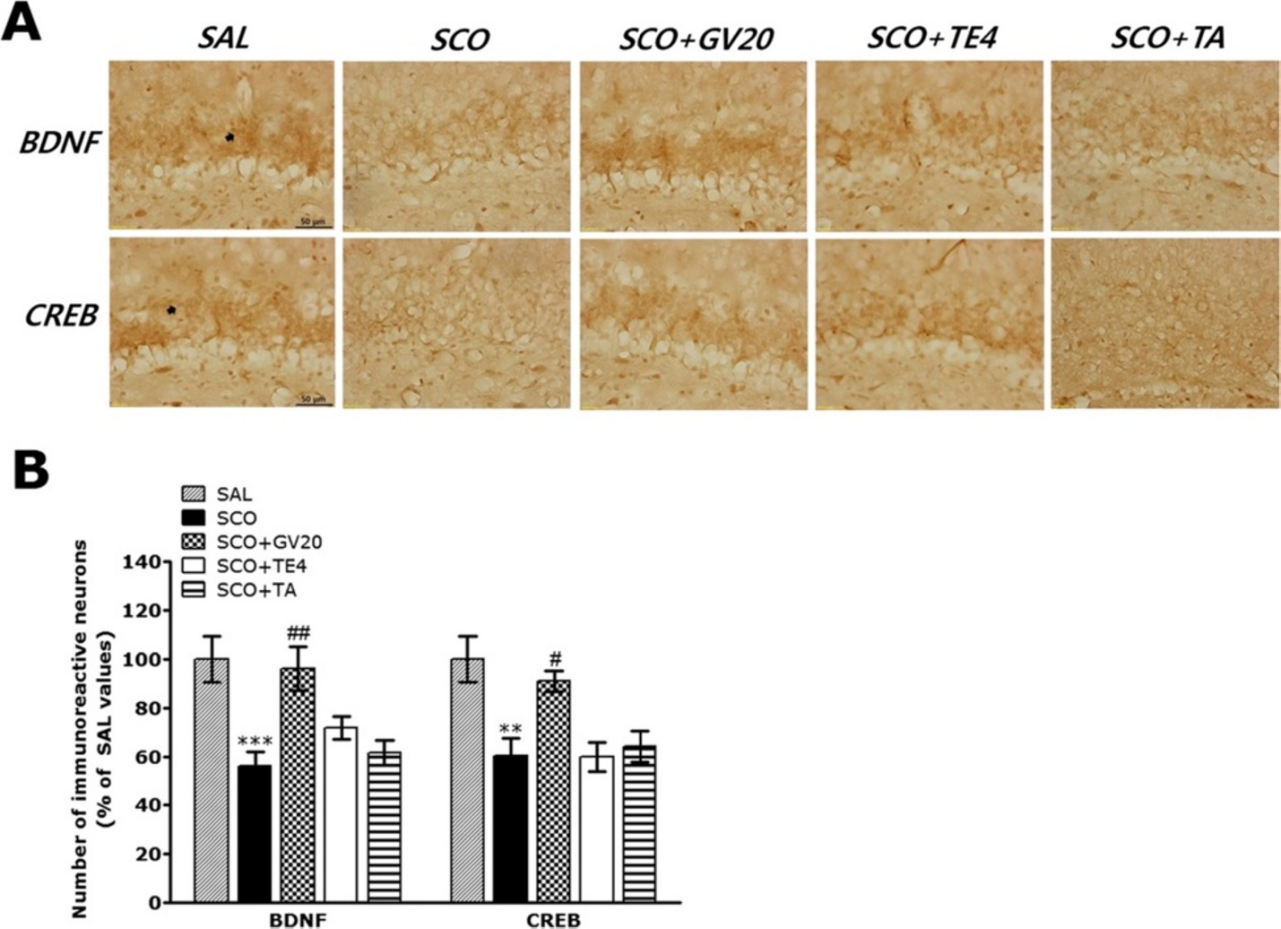
**Effect of GV20 acupuncture stimulation on the percentage values of the mean (±standard errer, SE) number of brain-derived neurotrophic factor (BDNF) and cAMP-response element-binding protein (CREB)-stained neurons in various hippocampal area after the Morris water maze (MWM) task (n = 3-4/group).** Representative photographs and relative percentage values are shown in **(A)** and **(B)**, respectively. (a): BDNF expression in the SAL group, (b) BDNF expression in the SCO group, (c) BDNF expression in the SCO + GV20 group, (d) CREB expression in the SAL group, (e) CREB expression in the SCO group, (f) CREB expression in the SCO + GV20 group. Sections were cut coronally at 30 μm. Scale bar represents 50 μm. ^**^ *p* < 0.01, ^***^ *p* < 0.001 *vs.* the SAL group; ^#^ *p* < 0.05 and ^##^ *p* < 0.01 *vs.* the SCO group.

Additionally, results of BDNF and CREB immunoreactivity analysis in the hippocampus are shown in Figure 6A. The number of BDNF-immunopositive neuronal cells in the rat hippocampus in the SCO group was significantly decreased compared with that in the SAL group (*p* < 0.001; Figure 6B). The BDNF-reactive neuronal activity in the hippocampus was significantly increased in the hippocampal region in the SCO + GV20 group compared with that in the SCO group (*p* < 0.01). The results also indicated that the SCO-induced rats treating acupuncture stimulation of GV20 was significantly increased BDNF expression in the hippocampal region compared with those in the SCO + TE4 group and the SCO + TA group. Also, the number of CREB-immunopositive neuronal cells in the hippocampus in the SCO group was significantly decreased compared with that in the SAL group (*p* < 0.01; Figure 6B). The CREB-reactive neuronal activity in the hippocampus associated with SCO-induced memory impairment was significantly restored in the SCO + GV20 group compared with that in the SCO group (*p* < 0.05). The results also indicated that the SCO-induced rats treating acupuncture stimulation of GV20 was significantly increased CREB expression in the hippocampal region compared with those in the SCO + TE4 group and the SCO + TA group. This also indicated that the expression of ChAT, BDNF and CREB-immunopositive neuronal cells in the hippocampus of rats treating acupuncture stimulation of GV20, but not of TE4 or the tail, restored memory impairment-related cholinergic function, and activated BDNF and CREB expression.

### Effect of acupuncture stimulation of GV20 on SCO-induced expression of CHT1, VAChT, mAChR-M1, BDNF and CREB mRNAs in the hippocampus

**Figure 7:**
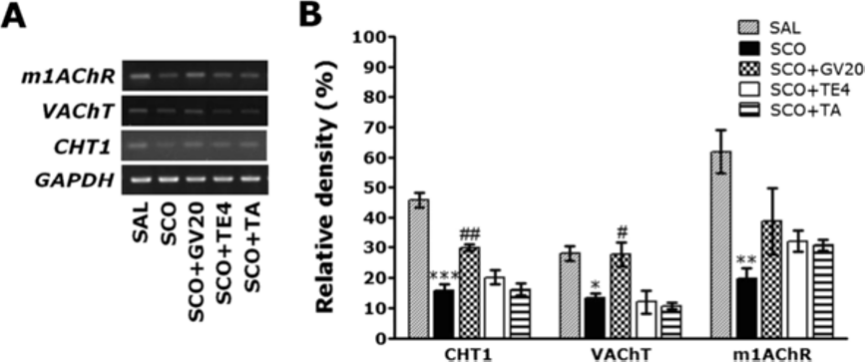
**Effects of GV20 acupuncture stimulation on the expression of vesicular acetylcholine transporter (VAChT), choline transporter 1 (CHT1) and muscarinic acetylcholine receptor type 1 (mAChR-M1) mRNAs in rats with SCO-induced hippocampal impairment (n = 3/group).** PCR bands on agarose gel and their relative intensities are shown in **(A)** and **(B)**, respectively. The expression levels of VAChT, CHT1 and mAChR-M1 mRNAs were normalized to that of glyceraldehyde 3-phosphate dehydrogenase (GAPDH) mRNA as an internal control. ^*^ *p* < 0.05, ^**^ *p* < 0.01, ^***^ *p* < 0.001 *vs.* the SAL group; ^#^ *p* < 0.05, ^##^ *p* < 0.01 *vs.* the SCO group.

**Figure 8:**
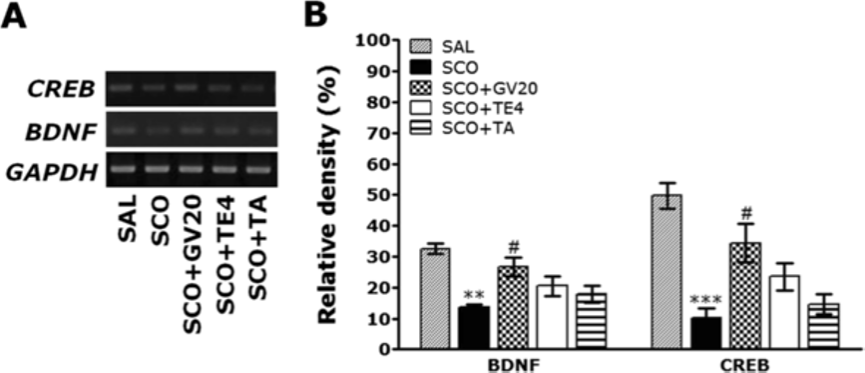
**Effects of GV20 acupuncture stimulation on the expression of brain-derived neurotrophic factor (BDNF) and cAMP-response element-binding protein (CREB) mRNA in rats with SCO-induced hippocampal impairment (n = 3/group).** PCR bands on agarose gel and their relative intensities are shown in **(A)** and **(B)**, respectively. The expression levels of BDNF and CREB mRNA were normalized to that of glyceraldehyde 3-phosphate dehydrogenase (GAPDH) mRNA as an internal control. ^**^ *p* < 0.05, ^***^ *p* < 0.001 *vs.* the SAL group; ^#^ *p* < 0.05 *vs.* the SCO group.

The effect of acupuncture stimulation at the GV20 on the expression levels of CHT1, VAChT, mAChR-M1, BDNF and CREB mRNAs in rats with SCO-induced hippocampus lesions was investigated using RT**-**PCR analysis (Figures 7 and 8). Hippocampal expression of CHT1 mRNA in the SCO group was significantly decreased compared with that in the SAL group (*p* < 0.001; Figure 7A). The decreased expression of CHT1 mRNA in the SCO group was significantly restored in the SCO + GV20 group (*p* < 0.01; Figure 7B). Hippocampal expression of VAChT mRNA in the SCO group was also significantly decreased compared with that in the SAL group (*p* < 0.05). The decreased expression of VAChT mRNA in the SCO group was significantly restored in the SCO + GV20 group (*p* < 0.05). Hippocampal expression of mAChR-M1 mRNA in the SCO group was significantly decreased compared with that in the SAL group (*p* < 0.01). The decreased expression of mAChR-M1 mRNA in the SCO group was not significantly restored in the SCO + GV20 group (*p* = 0.837).

Also, hippocampal expression of BDNF mRNA in the SCO group was significantly decreased compared with that in the SAL group (*p* < 0.01; Figure 8A). The decreased expression of BDNF mRNA in the SCO group was significantly restored in the SCO + GV20 group (*p* < 0.05; Figure 8B). Hippocampal expression of CREB mRNA in the SCO group was also significantly increased compared with that in the SAL group (*p* < 0.001). The decreased expression of CREB mRNA in the SCO group was significantly restored in the SCO + GV20 group (*p* < 0.05). This also indicated that the expression of CHT1, VAChT, BDNF and CREB mRNAs in the hippocampus in rats treating acupuncture stimulation of GV20, but not of TE4 or the tail, restored memory impairment-related cholinergic function, and activated BDNF and CREB mRNAs expression.

## Discussion

Our findings demonstrated that repeated SCO-induced memory impairment resulted in severe performance deficits in tests of cognitive functioning as well as corresponding signs of neurodegeneration in the brain, including decreased cholinergic enzyme activities and BDNF and CREB expression in the hippocampus. Our results showed that acupuncture stimulation at GV20 significantly improved learning and memory on the PAT, and decreased the escape latency and increased the number of platform crossings in the MWM test in a rat model of AD. Acupuncture stimulation also produced increased cholinergic enzyme activity, BDNF and CREB immunoreactivity, and increased CHT1, VAChT, BDNF and CREB mRNA expression in the hippocampus associated with SCO-induced memory impairment in male rats. Thus, these studies suggest that the effects of acupuncture stimulation at GV20 might be attributable to changes in behavioral task-related pathways that are modulated by the cholinergic system [30]. Further, coactivation of the cholinergic system and the BDNF-CREB pathway in the hippocampus might influence learning and memory processes, which may lead to development of novel therapeutics using acupuncture stimulation at GV20 to treat neurodegenerative diseases [31].

In traditional oriental medicine, the GV20 acupoint is one of the most important acupoints used to select the Governor meridians, and is located on the highest point of the head where all of the yang meridians meet [20, 26]. Thus, GV20 acupoint stimulation is effective therapeutically following stroke and in anxiety and cognitive disorders [32]. GV20 acupoint stimulation is also frequently used to treat psychological maladies, as it exerts general sedative and harmonizing effects [33]. The GV20 acupoint has a stimulatory effect on the CNS rather than merely a peripheral nerve; we believe that it stimulates the CNS to release neurotransmitters or neuromodulators into the brain [34]. As a comparable control we also stimulated the Yangji (TE4) acupoint, which is on a different (large intestine) meridian; and the triple-energizer channel, which is used to treat immune depression and pain/neuropathy of the arm [27, 35]. Interestingly, 5-min acupuncture stimulation prior to SCO administration was sufficient to modulate SCO-induced neurochemical and behavioral responses. In addition, only acupuncture stimulation at GV20 elicited significant responses, compared with stimulation of another acupoint on a different meridian, TE4, or of a non-acupoint on the tail. These results indicate that stimulation of the acupuncture point spreads rapidly throughout the body and its effect is highly point-specific, at least in terms of modulating SCO-induced memory impairments. Furthermore, acupuncture stimulation at GV20 attenuates a complex behavioral syndrome and protects the hippocampus from deficits [36–38].

Although some studies have aimed to elucidate the effects of acupuncture on neurodegenerative diseases, the effect of acupuncture on SCO-induced cognitive deficits and behavioral and neurochemical responses has not been reported.

To identify the effects of acupuncture stimulation at GV20 on two types of memory (cognitive memory and spatial learning) the PAT and MWM, respectively, were utilized. In the PAT, acupuncture stimulation at GV20 significantly increased step-through latency in the memory retention trial following chronic SCO administration. During MWM trial sessions, acupuncture stimulation at GV20 resulted in a significant reduction in escape latency, enhanced cognitive performance, and ameliorated the memory deficits associated with AD or dementia. These results indicate that chronic SCO administration to rat slows escape latency in the MWM test, which demonstrates deficits in spatial learning ability and reference memory [39]. The escape latency score determined by the MWM spatial probe tests is considered to reflect long**-**term spatial memory ability [39]. In the present study, acupuncture stimulation at GV20 shortened the escape latency without affecting swimming velocity, and extended the duration of swimming at the location where the platform was placed previously. This indicates that acupuncture stimulation at GV20 significantly improved long-term memory deficits in rats following chronic SCO-induced damage. Based on the results of the PAT and MWM tests, it can be postulated that chronic SCO-induced responses play an important role in learning acquisition and synaptic plasticity [40]. An open-field test was also performed to rule out any confounding motor impairments that can influence outcomes in many behavioral tests of depression. No significant individual differences in locomotor activity were observed between groups, suggesting that GV20 stimulation had no effect on sensorimotor performance. Accordingly, changes in behavioral performance in the MWM task were likely due to improved memory rather than differences in sensorimotor function, motor output, or limb flexibility.

The central cholinergic pathway is known for its critical role in learning acquisition and synaptic plasticity in the mammalian limbic system, and ACh is another important factor [41, 42]. By treating memory-related disorders with acupuncture stimulation at the GV20, the salvage capacity of ACh is enhanced (through enhanced CHT expression and activity, for instance); it also improves cholinergic neuron function in the fronto-parietal cortex and CA1 region of the hippocampus, and continuously induces increases in ChAT and AchE enzymatic activities, resulting eventually in recovery of the entire cholinergic system circulation pathway [15]. ChAT and AchE belong to the family of enzymes that are expressed at a high level in cholinergic neurons. ChAT is responsible for ACh biosynthesis and is required for cholinergic neurotransmission in the central and peripheral nervous systems [43]. Because ACh is rapidly hydrolyzed by AchE, the duration of ACh action in the synaptic cleft is dependent on AchE activity [42]. According to the cholinergic hypothesis, memory impairment in patients with senile dementia is due to selective and irreversible deficits in cholinergic function or alternation in hippocampal functioning in the brain [41, 43]. The expression and activation of AchE and ChAT regulate the dynamic concentration of ACh in the cholinergic synapses in AD brain [41]. Thus, we propose that the beneficial effects of acupuncture stimulation at GV20 in ameliorating memory impairment could be related to an increase in central cholinergic functioning. We also demonstrated a significant decrease in CHT1, VAChT and mAChR-M1 mRNA levels expression in rat hippocampal tissue following SCO-induced memory impairment in male rats, and acupuncture stimulation at GV20 induced an increase in CHT1 and VAChT mRNA levels, which might contribute to increased cholinergic activity. This study strongly suggests a direct correlation between reduced CHT1, VAChT and mAChR-M1 mRNA levels in the hippocampus and impaired cognition, which supports some of the results of the work presented brain [44]. The majority of ACh is rapidly hydrolyzed by AchE to choline. High-affinity CHT1 recycles choline from the synaptic cleft back to presynaptic terminals for ACh resynthesis [45]. Of the orchestrated events leading to the regulation of ACh release and cholinergic neurotransmission, the rate-limiting step is the recycling of choline by CHT [46]. In the presynaptic terminals, CHTs, present in endosomes, predominantly localize in synaptic vesicles [46]. Also, VAChT packages newly formed ACh into synaptic vesicles to prepare its release to the synaptic cleft. VAChT activity may affect generation of the readily releasable ACh pool [47, 48]. Specifically, in neurons that possess small releasable pools of synaptic vesicles, including central cholinergic neurons, the rate of filling recycling vesicles may be directly influenced by the level of VAChT [37, 48]. Therefore, new findings concerning the use of CHT1 and VAChT activation in the brain to develop novel therapeutics for healing memory deficits have been reported in several basic and clinical studies [49]. Also, mAChRs are G protein-coupled receptors that mediate the actions of the neurotransmitter, acetylcholine [50]. Although ubiquitously expressed in the CNS, the highest M1 protein expression is found in the cortex, hippocampus, and striatum, both presynaptically and postsynaptically [50]. Some studies suggest that mAChR-M1 receptors have a number of functions, including in learning and memory, and are implicated in a number of human diseases, including AD and dementia [50]. Thus, M1 agonists, in addition to their expected use as a cholinergic replacement strategy, might be of unique value in delaying the progression of memory deficits due to repeated SCO injection in male rats [51]. Therefore, chronic SCO-induced memory deficits result in significant reductions in mAChR-M1 expression in the hippocampus and poor performance on hippocampus-dependent tasks [50]. However, our results showed significant decreases in mAChR-M1 mRNA expression in rat hippocampal tissue following SCO-induced memory deficits and showed that acupuncture stimulation at GV20 did not significantly restore mAChR-M1 mRNA levels. Despite the lack of statistical significance, acupuncture stimulation at GV20 resulted in a trend toward recovery of the mAChR-M1 mRNA level. The reason for the observed differences in mAChR-M1 mRNA levels following GV20 acupuncture stimulation of cholinergic neuron action produced by SCO-induced memory impairment in male rats also should be investigated further.

Many studies strongly support the role of BDNF in modulating of synaptic function and plasticity in the CNS during learning and memory processes in addition to its actions related to neuronal cell survival and prevention of neurodegeneration [52]. Further, sufficient evidence exists to indicate that CREB regulates the expression of genes involved in neuroplasticity, cell survival, and long-term memory formation [53, 54]. Thus, BDNF transcription, regulated by CREB, may also be a critical player in the adaptive neuronal responses underlying learning and memory function [12]. Thus, a previous study suggested a close correlation between reduced expression of BDNF and CREB in the hippocampus with cognitive impairment [11]. SCO-induced memory deficits were associated with significant reductions in BDNF and CREB expression in the hippocampus and disruption in hippocampal function during working memory [9, 14]. We thus propose that acupuncture stimulation at GV20 significantly prevented SCO-induced reductions in BDNF and CREB expression. These findings suggest that the beneficial effects of acupuncture stimulation at GV20 include decreased SCO-induced memory and learning deficits due to increased BDNF expression via the CREB signaling pathway and could be related to increased neuronal functioning [14]. We also demonstrated significantly decreased BDNF and CREB mRNA levels in rat hippocampal tissue following SCO-induced memory deficits, and showed that acupuncture stimulation at GV20 restored the BDNF and CREB mRNA levels. This study strongly suggests a close correlation between reduced BDNF and CREB protein levels and gene expression in the hippocampus.

According to our results, acupuncture stimulation at GV20 in ameliorating memory impairment could be related to an increased in central cholinergic functioning. Thus, we concluded that the effect of acupuncture stimulation at GV20 is interrelated with primary alternation of ChAT and AchE activities as changes of the cholinergic system, furthermore, there are several major signaling pathways implicated in learning and memory [55]. Therefore, we also demonstrated that the levels of BDNF and CREB expression in the hippocampus involved after acupuncture stimulation with SCO to investigate the mechanism of the modulating action of the cholinergic system. Our results have shown altered BDNF and CREB expression levels in the hippocampus with dementia in rats, accompanied by the impairment of cognition in behavioral tests, which might be attributed to the reduction of cholinergic activity [56]. Some studies suggested that the cholinergic system acted on hippocampal newborn cells via CREB signaling [15]. The cholinergic system might activate the CREB signaling via a change of the BDNF level in the hippocampus. BDNF is known to enhance the phosphorylation of CREB and the survival of newborn cells [15]. In the present study, SCO reduced the BDNF level in hippocampus, consistent with the previous studies that the deficiency of central cholinergic system reduced the level of hippocampal BDNF mRNA [6]. Therefore, our results suggested that cholinergic activation by acupuncture stimulation regulated the mRNA of BDNF in the hippocampus. Taken together, we suggested that acupuncture stimulation might have the effect on the preserving of neuronal activity of the CREB-regulated BNDF pathway in hippocampal cholinergic neurons by restoring ACh level.

Acupuncture improves reversible malfunctions of the body via direct activation of various brain pathways and thus contributes to the restoration of normal systemic balance, probably due to regulation of neurotransmitters, including Ach [30]. Currently, acupuncture is a relevant complementary and alternative therapy for managing various cognitive disorders and psychosomatic diseases, such as stress, ischemia, and dementia [55]. Several studies demonstrated that electroacupuncture stimulation (EA) at GV20 and Dazhui (GV14) improves motor recovery, and stimulates BDNF/trkB expression in rats with focal cerebral ischemia [14, 57]. Acupuncture stimulation at Shenmen (HT7), Zusanil (ST36), Fenglong (ST40) and Taixi (KI3) improves brain function in AD patients [58], and acupuncture stimulation at ST36 protects against cognitive impairment caused by cerebral multi-infarct dementia in rats [35]. Acupuncture stimulation at GV20 alleviates the spatial memory impairment induced by cerebral multi-infarction, as evaluated by shortened escape latency and increased swimming time in the target quadrant in rats [59]. Therefore, these findings suggest that acupuncture stimulation at GV20 can ameliorate memory-related performance in many behavioral tests as well as modulate cholinergic neurons and regulate BDNF and CREB expression, which supports some of the results of this study.

Some studies suggest that only 5-min acupuncture stimulation can improve memory function in animal models and it appears to have a therapeutic effect on several pathological disorders. For example, the long-term electroacupuncture at acupoints Baihui (DU20) and Zusanli (ST36) for 5 min relieves the increased mean arterial pressure (MAP) and cerebral abnormality in both structure and function in spontaneously hypertensive rats (SHR), this beneficial action is most likely mediated via inhibition of angiotensin II type I receptor (AT1R)-endothelin receptor (ETAR)-endothelin-1 (ET-1) pathway [56]. Also, acupuncture stimulation to the HT7 acupoint for 5 min significantly ameliorated learning and memory deficits through recovery of the acetylcholine system. Acupuncture improved performance on the spatial memory test and protected septohippocampal cholinergic neurons from exogenous corticosterone-induced destruction [24]. Therefore, we think that only 5-min acupuncture stimulation to GV20 acupoint clearly well elicited effective responses in learning and memory functions in our results. Our results indicated that only 5-min acupuncture to GV20 was capable of attenuating impairments of memory and cognition and protecting the hippocampus from deficits.

Our study is basically intended to test effect of acupuncture stimulation at GV20 on prevention of repeated SCO-induced memory impairment rather the cure. Therefore, in order to test acupuncture stimulation at GV20 as the therapeutic method for improve learning and memory in rats exposed to repeated SCO-induced memory deficits, the acupuncture stimulation at GV20 was bilaterally applied before the SCO injection, and thereby we did examine to evaluate the ability of acupuncture stimulation at GV20 on prevention as measured by their performance in the PAT and the MWM test. Many studies suggest that manual acupuncture treatment at Kunlun (BL60) acupoint before formalin injection showed significant inhibited not only flinching behavior in the late phase of the formalin test model but also c-Fos expression in the spinal dorsal horn [60]. Also, Yanggu (SI5) acupuncture for 1 min immediately prior to morphine injection has been shown to suppress the reinstatement of morphine-seeking behavior selectively through reducing the motivation for drug via GABA receptor system [61]. Therefore, our results showed that acupuncture stimulation at GV20 for 5 min immediately prior to SCO injection might be attributable to changes in behavioral task-related pathways that are modulated by the cholinergic system.

## Conclusion

The present study demonstrated that the cognitive deficits and memory impairment observed after SCO-induced hippocampal lesion are closely related to the degeneration of cholinergic neurons and the BDNF-CREB pathway; acupuncture stimulation at GV20 significantly ameliorated spatial memory deficits through recovery of the ACh system. Moreover, the improvement in SCO-induced cognitive decline following acupuncture stimulation at GV20 could also be due to the alleviation of cholinergic neurochemical abnormalities and activation of BDNF and CREB expression. In conclusion, acupuncture stimulation at GV20 may be a useful alternative therapy for AD-type dementia.

## Abbreviations

GV20: Baihui
SCO: Scopolamine
BDNF: Brain-derived neurotrophic factor
CREB: cAMP-response element-binding protein
PAT: Passive avoidance test
MWM: Morris water maze
ChAT: Choline acetyltransferase
CHT1: Choline transporter 1
VAChT: Vesicular acetylcholine transporter
AD: Alzheimer’s disease
ACh: Acetylcholine
AChE: Acetylcholinesterase
CNS: Central nervous system
PD: Parkinson disease
PBS: Phosphate-buffered saline
ABC: Avidin-biotin-peroxidase complex
DAB: 3,3’-diaminobenzidine
mAChR-M1: Muscarinic acetylcholine receptor type 1
RT-PCR: Reverse transcription**-**polymerase chain reaction
GAPDH: Glyceraldehyde 3-phosphate dehydrogenase
ANOVA: Analysis of variance
TE4: Yangji
EA: Electroacupuncture stimulation
GV14: Dazhui
HT7: Shenmen
ST36: Zusanil
ST40: Fenglong
KI3: Taixi.
